# Supplementary material for: The causal relationship between the human gut microbiota and pyogenic arthritis: a Mendelian randomization study
Source: Front Cell Infect Microbiol. 2024 Nov 26;14:1452480. doi: 10.3389/fcimb.2024.1452480 (PMC11629706; doi:10.3389/fcimb.2024.1452480)
Supplement: Supplementary file 1 [file DataSheet1.zip › mendelian test/ebi-a-GCST90027490.csv_leave_one_out.pdf]

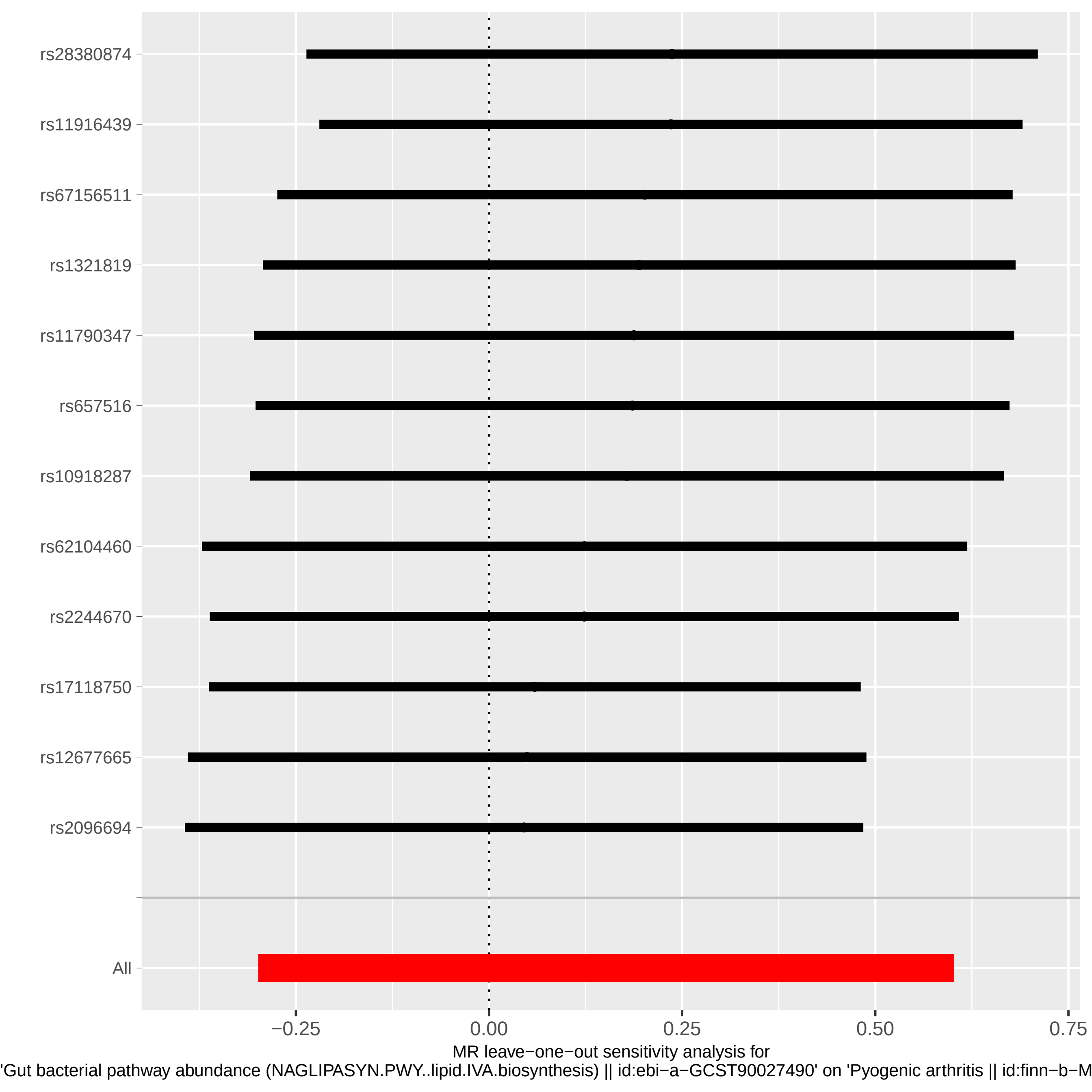
rs28380874
rs11916439
rs67156511
rs1321819
rs11790347
rs657516
rs10918287
rs62104460
rs2244670
rs17118750
rs12677665
rs2096694
All
−0.25
0.00
0.25
0.50
0.75
MR leave−one−out sensitivity analysis for
'Gut bacterial pathway abundance (NAGLIPASYN.PWY..lipid.IVA.biosynthesis) || id:ebi−a−GCST90027490' on 'Pyogenic arthritis || id:finn−b−M
